# Gasotransmitters bridging tumor biology and immunity: from pathophysiological insights to therapeutic potential

**DOI:** 10.3389/fimmu.2026.1671203

**Published:** 2026-01-28

**Authors:** Giulia Ballerini, Andrea Balboni, Valentina Garlatti, Martina Incerti, Antonio Sica, Francesca Maria Consonni

**Affiliations:** 1 Department of Pharmaceutical Sciences, University of Piemonte Orientale “A. Avogadro”, Novara, Italy; 2 IRCCS Humanitas Research Hospital, Milan, Italy

**Keywords:** gasotransmitters, immunosoppression, metabolism, myeloid cells, tumor micreoenvironment (TME)

## Abstract

The tumor microenvironment (TME) is a highly intricate and dynamic milieu, comprising neoplastic, immune and stromal cells in concert with extracellular matrix components, all engaged in continuous bidirectional crosstalk that critically orchestrates disease progression and therapeutic resistance. Beyond the local context, the TME is deeply shaped also by systemic influences, such as inflammatory mediators, metabolic cues and hematopoietic perturbations, collectively fostering a tumor-permissive macroenvironment. The interplay between local and systemic signals plays a pivotal role in modulating cellular differentiation, immune dynamics and stromal architecture, thereby sustaining malignancy. Among the myriad regulatory modulators involved in this complex network, endogenously produced gasotransmitters, namely carbon monoxide (CO), nitric oxide (NO) and hydrogen sulfide (H2S), have emerged as key modulators of tumor biology. These small, diffusible molecules exert a context-dependent spectrum of both pro-and anti-tumorigenic effects, influenced by their concentration, cellular source and tumor-specific microenvironmental conditions. Through the modulation of redox balance, metabolic signaling and epigenetic regulators, gasotransmitters impact immune cell functions, stromal remodeling and tumor cell behavior, thereby contributing to either immune evasion and therapy resistance or, conversely, to tumor suppression. Despite their growing relevance, the molecular mechanism governing these dualistic roles remain incompletely elucidated. This review provides a comprehensive overview of the current knowledge regarding the roles of CO, NO and H_2_S in shaping TME. We focus on their influence on immune, stromal and tumor cell differentiation, metabolism and function, and discuss how this understanding could inform novel therapeutic strategies aimed at reprogramming the TME to enhance clinical outcomes in cancer treatment.

## 1 Introduction

Resistance to therapy and metastatic dissemination remain the principal causes of cancer-related mortality (1). These processes are driven by the complex and dynamic nature of the TME, a highly interconnected network composed of tumor, immune, stromal and endothelial cells, as well as extracellular matrix components (2). In addition to the effects of inflammatory mediators released during tumor progression, the metabolic reprogramming and intense competition for essential nutrients, such as amino acids, glucose, fatty acids, and oxygen, between proliferating cancer cells and infiltrating immune cells profoundly reshape their metabolic states and functional behaviors (3). At both the primary tumor sites and distant metastatic niches, continuous and reciprocal interactions among these cellular and molecular players actively shape disease evolution (4). Importantly, the TME extends beyond the local tumor site, integrating systemic signals that influence immune homeostasis, haematopoiesis and the preconditioning of future metastatic sites (5, 6). Together, these local and systemic networks form an integrated macroenvironment that support tumor progression and foster immune suppression, thereby limiting the efficacy of both conventional and immune-based therapies.

A critical feature of this systemic dysregulation is emergency myelopoiesis, a stress-driven hematopoietic reprogramming induced by tumor-derived factors such as IL-1β, IL-6, GM-CSF, and G-CSF (7, 8). This process impairs myeloid differentiation in the bone marrow, promoting expansion and mobilization of pathological myeloid populations, including myeloid-derived suppressor cells (MDSCs) and tumor-associated macrophages (TAMs) (9). These immunoregulatory cells infiltrate the tumor, where their functional heterogeneity and plasticity contribute to tumor growth, immune evasion and therapy resistance (10–12). Within this complex regulatory framework, endogenous gaseous transmitters carbon monoxide (CO), nitric oxide (NO) and hydrogen sulfide (H_2_S), have emerged as crucial modulators of both local and systemic tumor processes. These small diffusible molecules participate in regulating redox homeostasis, angiogenesis, immune cell differentiation, metabolic adaptation and stromal remodeling, exerting context-dependent pro- or antitumor effects (13, 14). For example, low concentrations of NO facilitate immune evasion and tumor growth by promoting angiogenesis and suppressing cytotoxic immune responses, whereas high levels of NO induce oxidative stress and tumor cell apoptosis (15). Similarly, CO modulates macrophage polarization and T cell activity, influencing immunosuppressive circuits within the TME (16, 17). H_2_S plays dual roles by reprogramming cancer cell metabolism and modulating inflammation, with implications for tumor progression and therapy resistance (18).

Importantly, gaseous transmitters also influence the differentiation and function of myeloid populations expanded by emergency myelopoiesis, thereby modulating the immunosuppressive landscape of the TME (13). Their ability to modulate signaling pathways involved in inflammation, oxidative stress and cellular metabolism underscores their potential as therapeutic targets (19). Harnessing or inhibiting these gasotransmitter pathways may offer novel strategies to disrupt tumor-promoting microenvironmental cues and enhance the efficacy of cancer treatments (14).

In this review, we comprehensively analyze the multiple roles of CO, NO and H_2_S in shaping the TME and systemic immune responses. We focus on their impact on myeloid cell biology, stromal interactions and tumor progression, with the aim of providing insights into how modulating gasotransmitter signaling pathways could overcome tumor-host interaction barriers and improve therapeutic outcomes in cancer patients.

## 2 Hydrogen sulfide

### 2.1 Endogenous sources of H_2_S in the TME

H_2_S biosynthesis within the TME is a spatially regulated and cell-type–specific process occurring through both enzymatic and non-enzymatic pathways (20). Endogenously, H_2_S is produced primarily in mammalian cells by three pyridoxal phosphate (PLP)-dependent enzymes—cystathionine β-synthase (CBS), cystathionine γ-lyase (CSE), and 3-mercaptopyruvate sulfurtransferase (3-MST)—as well as the mitochondrial cysteinyl-tRNA synthetase 2 (CARS2) (21–23). CBS and CSE utilize L-cysteine and homocysteine as substrates, whereas 3-MST acts on 3-mercaptopyruvate, itself derived from L-cysteine via cysteine aminotransferase (20). These enzymes show heterogeneous expression across various cellular components of the TME, including malignant epithelial cells, stromal fibroblasts, endothelial cells and diverse immune subsets, thereby imparting a high degree of spatial and functional specificity to H_2_S-mediated signaling (13, 24).

CBS and CSE have been found to be up-regulated across different malignancies, with their expression levels correlating with more aggressive tumor phenotype (25, 26). CBS, initially characterized in hepatic and neuronal tissues, has been shown to localize in both the cytoplasm and mitochondria of cancer cells (21). CBS expression is markedly upregulated in colon (27), ovarian (28) and breast cancer cells (29), leading to enhanced endogenous H_2_S production compared to adjacent normal tissues or non-malignant cell lines. Its upregulation promotes mitochondrial ATP production through persulfidation of ATP synthase, facilitating metabolic reprogramming in support of proliferative and angiogenic signaling (30). In line, *in vivo* silencing of CBS in colon cancer xenograft models results in significant tumor regression, reduced microvascular density and increased oxidative stress, highlighting its role as a critical metabolic oncogene (27). Similarly, CSE is expressed not only in malignant cells but also abundantly in cancer-associated fibroblasts (CAFs), tumor-associated endothelial cells (TECs), and vascular smooth muscle cells, where it facilitates vasodilation, extracellular matrix remodeling, and neovascularization (31). Hypoxia, a defining feature of the TME, induces CSE expression through HIF-1α–mediated transcription, enhancing local H_2_S availability and sustaining stromal adaptation (32).

3-MST, which operates in conjunction with cysteine aminotransferase (CAT) to convert 3-mercaptopyruvate into H_2_S, is primarily localized to mitochondria and is particularly enriched in TECs and perivascular cells under hypoxic or nutrient-depleted conditions (33). Though its baseline expression is low in normal tissues, 3-MST is upregulated in several tumor types, including colon cancer (34), brain gliomas (35), lung adenocarcinomas (36) and renal cancer (37), where it supports oxidative phosphorylation, redox homeostasis and cell survival (38).

The most recently characterized H_2_S-producing enzyme, CARS2, encoded on chromosome 13q34, is a conserved mitochondrial enzyme primarily responsible for the aminoacylation of tRNA^Cys^ during mitochondrial protein synthesis (22). Beyond this canonical role, CARS2 has been identified as a non-canonical source of H_2_S, particularly under conditions of endoplasmic reticulum stress, amino acid deprivation, and integrated stress response (ISR) activation (39). Its expression is elevated in cancer stem-like cells and in aggressive malignancies such as hepatocellular carcinoma, colorectal cancer and basal-like breast cancer (40). CARS2 also participates in mitochondrial biogenesis, apoptosis regulation and cellular stress adaptation, underscoring its multifaceted role in tumor biology (18). Although the mechanisms linking CARS2-derived H_2_S to cancer progression remain incompletely defined, its emerging functions highlight its potential as a therapeutic target (39).

Beyond malignant and stromal cells, recent evidence highlights that immune cells within the TME are active contributors to local H_2_S production (39). TAMs express both CBS and CSE, particularly when polarized toward an M2-like immunosuppressive phenotype (41, 42). In these cells, H_2_S promotes anti-inflammatory cytokine production, such as IL-10, and upregulates PD-L1 expression, thereby facilitating immune evasion.

Dendritic cells (DCs) also express CSE and generate H_2_S in response to inflammatory and hypoxic stimuli, which skews them toward a tolerogenic state characterized by diminished antigen-presenting capacity and impaired IL-12 secretion (43). MDSCs, another immunoregulatory population within tumors, rely on CBS and CSE-derived H2S for maintaining their suppressive function (44). Moreover, in gastrointestinal and oropharyngeal cancers, microbiota-resident sulphate-reducing bacteria (SRBs) contribute an additional exogenous source of H_2_S (45, 46). Bacterial H_2_S has been shown to diffuse into the mucosa and affect immune homeostasis through the modulation of oxidative stress, toll-like receptor (TLR) signaling, and recruitment of regulatory immune cells, adding another layer of complexity to the local H_2_S landscape (46, 47).

### 2.2 H_2_S-mediated modulation of immune responses in cancer

H_2_S exerts multifaceted immunomodulatory effects within the TME, acting through both direct signaling mechanisms and redox-dependent events, in a highly context- and concentration-dependent manner (18). While low physiological levels can support immune activation under non-pathological conditions, in the cancer setting elevated intratumoral H_2_S levels, often driven by upregulation of CBS and CSE in cancer, stromal and immune cells, are predominantly immunosuppressive and facilitate tumor immune evasion (14, 39).

Experimental evidence has revealed that high concentrations of H_2_S compromise the survival and cytotoxicity of CD8^+^ cytotoxic T lymphocytes (CTLs) and natural killer (NK) cells. *In vitro* treatment with NaHS, a fast-releasing H_2_S donor, induces dose-dependent inhibition of lymphocyte proliferation via necrosis associated with mitochondrial depolarization and ΔΨm loss, sparing CD4^+^ T cells. Co-treatment with reduced glutathione rescues this effect, implicating oxidative imbalance as a key mediator of H_2_S-induced immunotoxicity (48). In breast cancers, co-culture assays using MDA-MB-231 cancer cells have clearly shown that silencing CBS and CSE leads to significant restoration of NK cell–mediated cytotoxicity; specifically, CBS/CSE knockdown upregulated NK-activating ligands on tumor cells, resulting in enhanced immune-mediated clearance *in vitro* (49).

Furthermore, H_2_S critically contributes to the expansion and function of Foxp3^+^ regulatory T cells (Tregs) (50). Mechanistically, a seminal study by Yang et al. demonstrated that H_2_S stabilizes Foxp3 expression and promotes Treg lineage commitment via persulfidation of NFYB, which upregulates Tet1/2 expression. These enzymes mediate active demethylation of the Foxp3 locus, a process further facilitated by Smad3 and STAT5 recruitment, under TGF-β and IL-2 signaling, respectively, reinforcing the transcriptional activity of Foxp3 in a H_2_S-dependent manner (50). In colorectal cancer-bearing mice, genetic knockdown or pharmacological inhibition of CBS and CSE resulted in a marked decrease in CD4^+^CD25^+^Foxp3^+^ Treg frequencies, both in spleen and tumor tissue, and concomitantly increased the CD8^+^/Treg ratio (51).

Beyond modulating adaptive immunity, H_2_S critically shapes innate immune cells in the TME (14). It drives macrophage reprogramming toward an immunosuppressive M2-like phenotype via redox-sensitive signaling, with CBS and CSE upregulated by hypoxia and cytokines (TGF-β, IL-6) (18, 41). In LPS-activated RAW264.7 macrophages, slow-releasing H_2_S donors (JK1, GYY4137) suppress NF-κB–mediated pro-inflammatory responses (iNOS, TNF-α, IL-6) while promoting M2 markers (Arg1, CD206) through STAT6 activation (52, 53). In THP-1 macrophages, GYY4137 stabilizes HIF-1α and activates the Nrf2/HO-1 pathway, further inhibiting NF-κB–dependent cytokine production (54). Silencing CSE reduces NF-κB activity and Glut1 expression, showing that endogenous H_2_S sustains macrophage metabolism and inflammatory responses via NF-κB and PI3K/Akt, dampening M1 polarization and glucose consumption (55–57). Importantly, slow-release donors like GYY4137 inhibit proinflammatory mediators while boosting IL-10, whereas fast-release donors (NaHS) elicit biphasic effects, indicating that H_2_S outcomes depend on both concentration and release kinetics (53).

H_2_S also modulates MDSC functions. In a syngeneic murine melanoma model, treatment with the slow-releasing H_2_S donor diallyl trisulfide (DATS) suppressed both monocytic and granulocytic MDSC activity in the spleen and TME, reducing tumor growth and systemically lowering MDSC frequency in spleen, blood, and tumor. DATS also promoted expansion of dendritic cells and CD8^+^ T cells in the spleen, although tumor infiltration was not increased (44). This immunological reprogramming involved transcriptional downregulation of immunosuppressive genes in MDSCs, restoring T cell proliferation (58). These findings illustrate the dual role of H_2_S in cancer therapy, mediating both anti-inflammatory and antitumor effects. When combined with photothermal therapy (PTT), H_2_S mitigated the pro-inflammatory response typically induced by PTT, lowering TNF-α, IL-6, and IL-1β levels (58). This anti-inflammatory effect can enhance therapy by limiting tissue damage and preserving immunogenic potential. Overall, H_2_S acts as a context-dependent modulator of tumor immunity (**Table 1**), with effects shaped by local concentration, cell source and redox state, emphasizing the need for precise regulation rather than indiscriminate inhibition to therapeutically reshape the TME.

**Table 1:** H_2_S–mediated regulation of the tumor microenvironment.

H_2_S role	Cell types	Molecular mechanisms	Experimental models	References
Pro-tumoral	Cancer cells (CRC, breast, ovarian, NSCLC)	CBS/CSE upregulation → ↑ H_2_S → ATP synthase & LDHA persulfidation → ↑ OXPHOS & glycolysis	In vitro; in vivo (CRC xenografts)	(27–30, 36, 59–61)
	Cancer cells (prostate, NSCLC, HCC)	HIF-1α and NF-κB activation → EMT, ↑ MMP-2/9, invasion	In vitro; in vivo	(62–64)
	Endothelial cells / TECs (HUVECs)	VEGFR2–mTOR, PI3K/AKT/eNOS → angiogenesis	In vitro; in vivo	(27, 65)
	Macrophages (RAW264.7; THP-1-derived TAMs) and MDSC	CBS/CSE → M2 polarization, ↑ IL-10, ↑ PD-L1, ↑ immunosuppression	In vitro; in vivo	(41, 42, 44, 52–55)
	Cancer cells (CRC)	GPX4 upregulation, xCT stabilization → ferroptosis resistance	In vitro; in vivo	(66)
Anti-tumoral	Cancer cells (CRC, NSCLC)	CBS/CSE inhibition or H_2_S scavenging → ↑ ROS, ↓ ATP, tumor regression	In vitro; in vivo	(27, 36, 66)
	Epithelial tumor cells (gastric, CRC, breast, pancreatic)	GYY4137/SPRC → ↓ Smad2/3, Wnt/β-catenin → EMT suppression	In vitro; in vivo	(49, 51, 67–69)
	Innate immune cells (NK, DCs)	CBS/CSE silencing → ↑ NK cytotoxicity, ↓ tolerogenic DCs	In vitro; in vivo	(43, 50, 51)
	Cancer cells (NSCLC)	H_2_S-mediated homocysteine accumulation → oxidative stress → ferroptosis	In vitro	(70)

This table summarizes the pro- and anti-tumoral roles of H_2_S in the TME, reporting the involved cell types, tumor models, key molecular mechanisms, experimental settings and key references. The data highlight the pleiotropic and concentration-dependent functions of H_2_S in regulating cancer cell metabolism, angiogenesis, apoptosis and immune cell behavior within the TME.

### 2.3 H_2_S-dependent modulation of non-immune cellular dynamics in TME

H_2_S exerts multifaceted and context-dependent regulatory effects on non-immune cellular components of the TME, modulating key processes that govern tumor progression, including angiogenesis, metabolic reprogramming, epithelial–mesenchymal transition (EMT), DNA repair, ferroptosis inhibition; these actions are frequently concentration-dependent and intricately modulated by the intracellular redox milieu (**Table 1**) (13). H_2_S modulates matrix remodeling and cell adhesion by regulating the expression of matrix metalloproteinases (MMPs), integrins and cadherins, all of which are pivotal to local invasion and metastasis (67). H_2_S delivered via various donors including S-propargyl-cysteine (SPRC), NaHS, erucin and HA-ADT, has been shown to inhibit cancer cell migration and invasion in multiple *in vitro* models (i.e. gastric, colorectal, breast, pancreatic and melanoma) and reduce lung metastasis *in vivo* (71). Pharmacological inhibition of CSE in A549 human alveolar epithelial cells elicits EMT, as evidenced by diminished E-cadherin expression and upregulation of mesenchymal markers (72); conversely, in epithelial tumor cells, exogenous H_2_S administration through GYY4137 prevent EMT by attenuating Smad2/3 phosphorylation and restoring E-cadherin expression (68). Similarly, H_2_S inhibits TGF- β1-induced EMT through the Wnt/β-catenin pathways, and modulation of MAPK signaling in a context-dependent manner (69).

Paradoxically, accumulating evidence also implicates H_2_S in the promotion of tumor invasiveness under specific pathological contexts (13). Endogenously generated H_2_S, predominantly via CBS, has been shown to facilitate migratory and invasive phenotypes in colorectal and non-small cell lung adenocarcinoma cells, effects that are mitigated upon enzymatic inhibition (27, 62). In these models, H_2_S-driven EMT, characterized by E-cadherin downregulation, vimentin upregulation and enhanced expression of MMPs, appears to be, at least in part, dependent on hypoxia-inducible factor-1α (HIF-1α), whose silencing effectively reverses these pro-metastatic changes (62).

Moreover, activation of the NF-κB signaling cascade by H_2_S has been implicated in the upregulation of MMP-2 and pro-inflammatory cytokines, thereby potentiating invasiveness in hepatocellular and prostate cancer models (63, 64). Intriguingly, contrasting findings have also documented an inhibitory role of H_2_S on NF-κB activity, underscoring the dualistic and context-dependent nature of its bioactivity within the TME (73). Within pathological contexts such as cancer, H_2_S serves predominantly as a facilitator of angiogenic processes (74).

Experimental models using NaHS and DATS have demonstrated that H_2_S activates the VEGFR2/mTOR and PI3K/AKT/eNOS pathways, leading to increased endothelial cell proliferation, migration and capillary-like tube formation (65). H_2_S also upregulates HIF-1α, further reinforcing the angiogenic cascade under hypoxic conditions (62). CBS silencing in colon cancer models markedly reduces micro vessel density and impairs tumor vascularization (27).

H_2_S contributes to chemoresistance by facilitating DNA repair and sustaining redox balance (75). It activates the ATR/CHK1 pathway, enhancing the DNA damage response (76), and, in lung adenocarcinoma models, increased H_2_S biosynthesis sustains mitochondrial DNA repair and bioenergetics; inhibiting H_2_S-producing enzymes sensitizes tumors to drugs like cisplatin (36).

Simultaneously, H_2_S enhances glutathione biosynthesis, raises intracellular GSH and upregulates GPX4, collectively protecting cells from ROS and ferroptosis (77). It inhibits ferroptosis by stabilizing the xCT antiporter, suppressing ALOX12, and increasing GPX4 (78). Interestingly, a zinc oxide nanosphere designed to scavenge endogenous H_2_S in colorectal cancer cells triggered ferroptosis and inhibited tumor growth (66). Conversely, in NSCLC it was recently reported that H_2_S through persulfidation mechanisms, disrupts homocysteine metabolism, leading to increased intracellular homocysteine and oxidative stress, which in turn sensitizes cells to ferroptosis (70), highlighting its context-dependent role as a ferroptosis suppressor or inducer. At the mitochondrial level, H_2_S serves as an alternative substrate for oxidative phosphorylation via SQR, enhances ATP synthase activity through persulfidation (Cys244/294), and increases mitochondrial cAMP by inhibiting PDE2A, activating PKA and boosting electron transport chain function (59, 60). Additionally, H_2_S persulfidates LDHA, stimulating glycolysis and reinforcing the shift toward oxidative glycolysis (61). This multifaceted enhancement of energy production confers survival advantages to cancer cells under metabolic stress, highlighting H_2_S as a critical modulator of mitochondrial function in the TME.

## 3 Carbon monoxide

### 3.1 Endogenous sources of CO in the TME

CO is endogenously produced in various mammalian cells and tissues by a family of enzymes known as heme oxygenase (HOs), which include three isoforms: HO-1, HO-2 and HO-3 (79). These enzymes catalyze the oxidative degradation of heme, generating CO along with ferrous iron (Fe^2+^) and biliverdin, the latter subsequently reduced into bilirubin (80). This enzymatic reaction is dependent on molecular oxygen and the reducing cofactor NADPH (80).

HO-1 is the inducible isoform and serves as the major enzymatic source of CO; it is mainly localized in the endoplasmic reticulum and displays a heterogeneous expression pattern across cell types, with particularly high levels in macrophages of the spleen, liver and bone marrow (17). Under basal physiological conditions HO-1 is expressed at low levels, but its expression is markedly increased in response to various cellular stressors and external stimuli (81). In contrast, HO-2 is a constitutive isoform expressed in multiple tissues, especially in the brain, kidney, liver and testis, where it plays physiological processes. HO-3 is also a constitutive isoform but lacks enzymatic activity towards heme and its biological function remains unclear (79). The HO-1/CO pathway is increased in response to oxidative stress, hypoxia and inflammatory cues, commonly found in several types of cancer, including esophageal, breast, gastric, colorectal, hepatic, pancreatic and prostate cancers, as well as neuroblastoma, and is often associated with poor clinical outcomes (81–83). CO exerts its biological effects mainly by binding to heme moieties in target proteins, modulating their activity and triggering intracellular signaling cascades that can be pro- or anti-inflammatory based primary on the CO concentrations and the environmental conditions (84).

Among its canonical targets, soluble guanylate cyclase (sGC) is activated upon CO binding, leading to increased production of cyclic guanosine monophosphate (cGMP), which modulates vascular tone, endothelial permeability and immune cell recruitment, inducing anti-inflammatory, anti-apoptotic and anticoagulant responses (85). Furthermore, at low concentrations, CO activates KATP channels and influences mitogen-activated protein kinases (MAPKs), particularly ERK1/2, JNK and p38, as well as the PI3K/AKT signaling axis, which collectively regulates cell proliferation, resistance to apoptosis and metabolic reprogramming (86). However, high concentrations of CO result in cytotoxicity, inducing mitochondrial dysfunction, loss of membrane potential, excessive ROS generation, cytochrome c release and intrinsic apoptosis (13, 87).

A variety of cell types are capable of producing endogenous CO through the activity of HO-1, including immune, stromal, endothelial and tumor cells (88). The induction of HO-1 in tumor cells is governed by transcription factors including nuclear factor erythroid 2–related factor 2 (NRF2), hypoxia-inducible factors (HIF-1α/2α), activator protein-1 (AP-1), nuclear factor kappa B (NF-κB) and signal transducer and activator of transcription 3 (STAT3) (81, 89). Notably, NRF2 activation following oxidative stress or electrophilic insults leads to its nuclear translocation and binding to antioxidant response elements (AREs) in the HO-1 promoter, resulting in enhanced transcription (90). Parallel activation of HIF-1α under hypoxic conditions synergistically upregulates HO-1, integrating redox and oxygen-sensing pathways to adapt the tumor to its microenvironmental constraints (89).

In addition to malignant cells, HO-1 is also expressed by DC, regulatory T cells, TAMs and endothelial cells (88, 91–93). Within the immune compartment, TAMs, particularly those polarized toward an M2-like, immunosuppressive phenotype, are the prominent sources of CO due to robust HO-1 induction (16, 94). In preclinical models of LL2 and PDAC, a distinct FAP^+^HO-1^+^ subset of M2-like F4/80^hi^ TAMs, comprising ~10% of total TAMs, has been identified as the main source of tumoral HO-1 (95). Similar populations have been found in human (96) and murine breast tumors (97). In the 4T1 breast cancer model, these perivascular FAP^+^HO-1^+^ TAMs promoted tumor cell migration and metastasis through wound-response cytokines and trans-endothelial support (97). Accordingly, a distinct subset of bone marrow–derived F4/80^hi^ HO-1^+^ TAMs was recently identified as key drivers of a pro-metastatic TME, promoting immunosuppression, angiogenesis, EMT and inhibition of T cell antitumor activity. These TAMs originate from circulating HO-1+ monocytes and localize to the invasive tumor margins (e.g., fibrosarcoma and melanoma) via the NF-κB1/CSF1-R/C3a pathway, which supports HO-1 expression (94). Even in the aggressive MMTV-PyMT breast cancer model, TAMs have been reported to represent the major source of tumoral HO-1 that sustains immunosuppressive pathways within the TME (98).

HO-1 expression has also been detected in dendritic cells (DCs), where it impairs their immunogenic function and antigen-presenting capacity (91). In addition, the immunosuppressive CD4^+^CD25^+^Foxp3^+^ regulatory T cell (Treg) subset expresses HO-1 in humans (99), suggesting a possible role for HO-1 within the Foxp3-regulated transcriptional program.

This pattern of HO-1 expression determines dynamic spatial and temporal variations in CO levels across the TME, underpinning the pleiotropic roles of this gaseous mediator in promoting tumor progression and immune evasion.

### 3.2 Immunomodulatory roles of CO in the TME

The production of CO by various cells population within the TME plays a critical role in modulating immune responses, influencing the function, polarization and survival of key immune populations involved in tumor progression and immune evasion in a concentration dependent manner (14).

Immunologically, low-level CO production, traditionally from 1 to 100 nM, skews TAMs toward an M2-like pro-tumoral phenotype, characterized by increased secretion of immunosuppressive cytokines such as interleukin-10 (IL-10) and transforming growth factor-beta (TGF-β) (100). This polarized state inhibits cytotoxic T lymphocyte (CTL) and natural killer (NK) cell functions, facilitating immune evasion. In line, in BM-derived macrophages, myeloid-specific deletion of HO-1 led to increased expression of pro-inflammatory markers (e.g. CXCL10, IL-1b and CCl2/MCP-1) following stimulation with polarizing signals such as LPS and IL-4, along with a concomitant reduction in anti-inflammatory markers (Arg1 and CD163) (101). Furthermore, following chemotherapy, TAMs upregulate HO-1 expression after phagocytosing tumor cell debris, which in turn impairs M1 polarization and compromises the overall efficacy of the therapy (102). Alaluf et al. also demonstrated that myeloid-specific ablation of HO-1 reduced Arg1 expression while increasing iNOS levels, and induced broad transcriptional and epigenetic changes in TAMs (103). Moreover, CO directly influence macrophage polarization *in vitro*, promoting an anti-inflammatory phenotype, through the modulation of TLR (104) and MAPK signaling pathways, leading to the downregulation of pro-inflammatory cytokines (TNF-α, IL-1β) and the upregulation of IL-10 (86). Consistently, *in vitro* exposure of macrophages to carbon-monoxide releasing molecules (CORMs), designed to deliver CO in a controlled manner and used as valuable experimental tools, enhances STAT3/STAT6 activation, further driving their polarization toward an anti-inflammatory state (94). These observations collectively demonstrate that the HO-1/CO axis dampens antitumor immunity and supports cancer progression through the formation of immunosuppressive TME (13).

Conversely, high CO concentrations, typically between 1 and 100 µM, modulate immune responses through an anti-tumoral phenotype by reversing M2-like macrophage polarization toward a pro-inflammatory M1-like phenotype, which secretes TNF-α, IL-12, and enhances cytotoxic T lymphocyte (CTL) and NK cell activity, collectively reinforcing antitumor immunity (105). The effects of high CO concentrations on macrophage polarization may be due, at least in part, to their ability to inhibit mitochondrial respiration and promote a shift in cellular metabolism toward glycolysis, a process more prominent in M1 macrophages. Accordingly, in the *in vivo* A549 lung carcinoma model, exposure of mice to exogenous CO induced macrophage polarization toward a pro-inflammatory M1-like phenotype via ROS-dependent activation of the MAPK/Erk1/2–c-Myc signaling pathway, contributing to an anti-tumor growth effect (105).

CO shapes an immunosuppressive tumor microenvironment through multiple mechanisms (106). It upregulates PD-L1 expression on tumor cells and antigen-presenting cells, via STAT3 and NF-κB, inhibiting T cells activation (14, 17). Furthermore, it promotes expansion and suppressive activity of Tregs by enhancing TGF-β production (107). In glioma patients, elevated HO-1 mRNA expression has been associated with increased Foxp3 induction in infiltrating CD4^+^CD25^+^ Tregs, correlating with tumor progression and higher glioma grade (92). Moreover, in preclinical breast cancer (4T1) and melanoma (B16) models, fasting-mimicking diet (FMD)-mediated HO-1 reduction in tumor cells, decreases Tregs activation, enhances infiltration of cytotoxic CD8^+^ T cells and sensitizes tumors to chemotherapy (108). CO also directly block T cells proliferation by inhibiting IL-2 secretion, ERK activation and inducing caspase-dependent growth arrest (109, 110). Paradoxically, CO can stimulate antitumor immunity through immunogenic cell death and DC maturation (111). The HO-1/CO axis maintains DCs in a tolerogenic state with increased IL-10 and reduced MHC II expression and suppresses NK cell function by downregulating activating receptors (NKG2D, NKp46, NKp30) and key cytokines (IFN-γ, TNF-α) (91, 112, 113). These findings highlight the dual immunosuppressive vs immune boosting effects of CO in TME depending on its concentration, cellular origin and signaling context (**Table 2**), making a deep understanding of these factors essential for therapeutic targeting of the HO-1/CO axis (13, 88).

**Table 2:** CO–mediated regulation of the tumor microenvironment.

CO role	Cell types	Molecular mechanisms	Experimental models	References
Pro-tumoral	Cancer cells (NSCLC, breast, CRC, pancreatic, melanoma, HCC)	HO-1 induction (NRF2, HIF-1α, STAT3, NF-κB) → CO production → ERK1/2 & PI3K/AKT activation, ↑ Bcl-2, cytoprotection	In vitro (human cancer cell lines); in vivo (xenografts, syngeneic models)	(81–83, 114–118)
	Cancer cells (various solid tumors: esophageal, breast, gastric, colorectal, hepatic, pancreatic, prostate, neuroblastoma)	CO-mediated stabilization of HIF-1α (PHD inhibition, ROS) → ↑ VEGF, glycolytic switch, hypoxia adaptation, ↑ sGC/cGMP, ↑ KATP channels and MAPKs	In vitro; in vivo	(85, 86, 119, 120)
	Cancer cells (mitochondria: hepatoma, pancreatic)	Mild inhibition of cytochrome c oxidase (Complex IV) → controlled ROS signalling → pro-survival pathways	In vitro	(121)
	Endothelial cells / TECs (HUVECs, tumor-derived Ecs; breast, pancreatic model)	sGC/cGMP → PI3K/AKT, MAPK/ERK, eNOS activation → proliferation, migration, angiogenesis	In vitro (HUVEC assays); in vivo (HO-1 overexpressing mice)	(74, 122–125)
	Macrophages (TAMs) (F4/80hi, FAP^+^HO-1^+^ TAMs)	Low CO (nM) → M2 polarization, ↑ IL-10, TGF-β, Arg1 → immunosuppression	In vitro; in vivo (LL2, PDAC, 4T1, melanoma models)	(94–98, 100–103)
	Immune cells (DCs)	HO-1/CO → tolerogenic DCs, ↓ MHC II, ↓ antigen presentation, ↑ IL-10	In vitro; in vivo	(91, 112)
	Tregs (CD4^+^CD25^+^Foxp3^+^)	HO-1/CO → ↑ TGF-β, Foxp3 induction, suppressive activity	Clinical (glioma); in vivo (4T1, B16)	(92, 99, 107, 108)
	NK cells	HO-1/CO → ↓ NKG2D, NKp30, NKp46, ↓ IFN-γ and TNF-α	In vitro; in vivo	(113)
	CAFs	CO/CORMs → modulation of NF-κB, STAT3, TGF-β/Smad → fibroblast reprogramming supporting tumor growth	In vitro	(27–121, 126–133)
Anti-tumoral	Macrophages	High CO (µM) → inhibition of mitochondrial respiration → glycolytic shift → M1 polarization (↑ TNF-α, IL-12)	In vivo (A549 lung carcinoma model)	(105)
	T cells	High CO → immunogenic cell death, ↑ DC maturation → enhanced CD8^+^ T cell responses	In vitro; in vivo	(110, 111)
	Cancer cells cervical, NSCLC, breast, pancreatic, medulloblastoma, hepatoma, ovarian)	High CO → mitochondrial collapse, ↑ ROS, cytochrome c release → intrinsic apoptosis	In vitro; in vivo	(13, 87, 108)
	Cancer cells (cervical, breast, hepatoma)	CO → caspase-9/-3 activation, ↑ p21/p27 → cell cycle arrest	In vitro	(87, 110)
	Endothelial cells	High CO → endothelial apoptosis → impaired angiogenesis	In vitro; in vivo	(125, 134)
	Cancer cells (targeted delivery)	Photoactivatable antibody–photoCORM → selective CO cytotoxicity	In vivo (ovarian cancer)	(130)

This table outlines the dual roles of CO in the TME, distinguishing between pro-tumoral and anti-tumoral activities. For each cell type, the table reports the tumors studied, the molecular pathways modulated by CO, the experimental settings (*in vitro* and/or *in vivo*), and the relevant references. The data highlight the contribution of the HO-1/CO axis to immune regulation, tumor cell survival, angiogenesis and immune evasion.

### 3.3 Effects of CO on cancer cells and TME non-immunological components

The ability of tumor cells to frequently exploit HO-1 upregulation as an adaptive mechanism to resist oxidative and chemotherapeutic stress correlates with enhanced malignancy and poor clinical outcomes in cancers such as non-small cell lung carcinoma (NSCLC), breast carcinoma and melanoma (114). The functional relevance of intratumoral CO is underscored by multiple studies demonstrating its cytoprotective and pro-survival roles in cancer, with evidence showing that CO can actively promote tumor progression by enhancing both cell proliferation and angiogenesis. CO-induced activation of ERK1/2 and PI3K/AKT pathways increases expression of anti-apoptotic proteins (e.g. Bcl-2), facilitating survival of cervical, breast and colon cancer cell lines (115, 116). These findings have been corroborated by additional studies employing CORM-2 in non-small cell lung cancer (117) and pancreatic cancer models (118). In line with its cytoprotective role, exogenous CO enhances resistance to apoptosis and TGF-β1-induced cell cycle arrest in tumor cells through inhibition of K^+^ channels in medulloblastoma (119) and ERK1/2-mediated Smad3 phosphorylation in hepatocellular carcinoma (127). Moreover, siRNA-mediated silencing of HO-1 impairs viability and proliferation of pancreatic (128) and hepatoma cancer cells (129), both *in vitro* and *in vivo*, enhancing apoptosis and underscoring the pro-tumorigenic role of HO-1-derived CO. Recently, antigen-specific delivery of CO using a photoactivatable antibody-photoCORM system has shown promising results in selectively delivering CO and inducing cytotoxicity against cancer cells in an ovarian cancer model (130).

Crucially, CO stabilizes HIF-1α, through inhibition of prolyl hydroxylases and promotion of ROS, thereby fostering angiogenesis via upregulation of VEGF and promoting the glycolytic switch essential for tumor survival under hypoxia (120). Concurrent activation of NRF2 downstream of CO signaling enhances antioxidant defenses, mitigating oxidative damage and sustaining tumor cell viability (13, 120). Likewise, mitochondria represent critical effectors of CO activity; indeed, at low concentrations, CO mildly inhibits cytochrome c oxidase (complex IV), resulting in controlled ROS production that acts as secondary messengers to activate pro-survival pathways in cancer cells (121).

CO within TME exerts also a complex and dualistic influence on CAFs which are pivotal in modulating tumor progression (131). Exposure to CO or CORMs has been shown *in vitro* to downregulate pro-fibrotic and pro-inflammatory genes, including TGF-β and α-SMA, while reducing the secretion of tumor-promoting cytokines (132). Mechanistically, this reprogramming involves modulation of key signaling pathways such as NF-κB, STAT3, and TGF-β/Smad, leading to diminished fibroblast contractility and a reduced capacity to support tumor cell proliferation and invasion (133). Moreover, endothelial cells exposed to low CO concentrations exhibit increased proliferation and migration, contributing to neovascularization and tumor perfusion (122). At nanomolar concentrations, CO acts as a potent pro-angiogenic factor by activating the soluble guanylate cyclase (sGC)/cyclic GMP (cGMP) signaling pathway, which subsequently triggers downstream effectors including PI3K/AKT and MAPK/ERK cascades (74, 123). This activation enhances endothelial nitric oxide synthase (NOS3) activity and promotes the expression of VEGF (123). *In vitro* experiments using human endothelial cells, such as human umbilical vein endothelial cells (HUVECs), treated with low doses of CO donors (e.g. CORM-2 and CORM-3), have consistently shown increased cell proliferation, migration and tube formation, hallmark processes of angiogenesis essential for tumor vascularization (124). Complementary *in vivo* studies using HO-1 overexpressing mouse models revealed enhanced neovascularization and accelerated tumor growth, whereas HO-1 knockout mice displayed impaired endothelial function and reduced angiogenesis, confirming the critical role of endogenous CO production (125).

Conversely, beyond a certain threshold, exogenous administration of CO can induce tumor suppressive effects. High CO levels exacerbate mitochondrial dysfunction by inhibiting mitochondrial cytochrome c oxidase (complex IV), a critical enzyme in the electron transport chain responsible for ATP production (87). This inhibition disrupts mitochondrial membrane potential, leading to excessive production of reactive oxygen species that surpass cellular antioxidant capacities, triggering apoptotic pathways (87). Elevated ROS levels cause oxidative damage, activating the intrinsic apoptotic cascade characterized by increased caspase activation, particularly caspase-9 and caspase-3 and cell cycle arrest mediated by upregulation of cyclin-dependent kinase inhibitors p21 and p27 (87). In endothelial cells, this process results in programmed cell death, reducing angiogenesis by impairing the formation and maintenance of new blood vessels essential for tumor growth (134).

## 4 Nitric oxide

### 4.1 Endogenous sources of NO in TME

NO is a highly reactive and diffusible free radical, endogenously produced and able to act as a key signaling molecule in various physiological and pathological contexts (135, 136). It is generated from L-arginine in the presence of O_2_ and NADPH by a family of nitric oxide synthase enzymes and modulates several cellular processes including vasodilation, neurotransmission, immune response, and cell survival. NO activates soluble Guanylyl Cyclase (sGC), leading to increased intracellular levels of cyclic guanosine monophosphate (cGMP), which in turn triggers downstream signaling cascades involving cGMP-dependent protein kinases (PKG), protein kinase C (PKC), and mitogen-activated protein kinases (MAPKs), ultimately influencing vascular tone and neuronal communication (137). NO is also fundamental for the S-nitrosylation of various proteins involved in cellular signaling, such as PEBP-1 and PCNA (138).

NO is a pathogenic factor in tumors and the effects depend on its concentration and the duration of the exposure (139, 140). Low levels of NO (1–200 nM) have been shown to facilitate tumor progression and cell proliferation (141). Endogenous NO can inhibit caspase activity, while NO/cGMP interaction inhibits cytochrome C release and increases BCL-2 expression (142). Ultimately, NO can also induce a hypoxic response under normoxic conditions in TME via inhibition of prolyl hydroxylase-mediated degradation of HIF-1α (143). Conversely, NO showed a tumoricidal activity at higher doses acting as a proapoptotic modulator and suppressing DNA synthesis and tumor cell metastasis (144). In biological systems, several deleterious NO-mediated effects arise from its concurrent production alongside oxygen-derived ROS (145). A critical pathway implicated in this oxidative and nitrosative stress involves the rapid reaction of NO with superoxide anion (O_2_^−^), yielding the potent oxidant peroxynitrite (ONOO^−^) (145). The cell-specific and context-dependent expression of NOS isoforms orchestrates a multifaceted NO signaling network within the TME, underscoring the dual and often paradoxical roles of NO as both a tumor-promoting and tumor-suppressing mediator, depending on its local concentration, temporal dynamics, and cellular origin (140).

NO is enzymatically synthesized by a family of NOS isoforms, which catalyze the five-electron oxidation of L-arginine to L-citrulline and NO, in the presence of molecular oxygen, NADPH, flavin adenine dinucleotide (FAD), flavin mononucleotide (FMN), tetrahydrobiopterin (BH_4_) and calmodulin (136). In particular, NOS is a large enzyme ranging in size from 135 to 160kDa, that possesses an N-terminal oxygenase domain, where the reaction takes place, and a C-terminal reductase domain, that supplies electrons for the reaction (146). Overexpression of NOS in human tumors has been correlated with an increase of malignancy and poor patient survival (30, 147).

NOS presents 3 isoforms with a 51-57% homology: neuronal NOS (nNOS or NOS1), inducible NOS (iNOS or NOS2) and endothelial NOS (eNOS or NOS3). NOS1 and NOS3 are constitutively expressed and are regulated in a calcium/calmodulin-dependent manner, producing transient, low concentrations of NO primarily involved in physiological signaling (136). In contrast, NOS2 is transcriptionally induced in response to pro-inflammatory cytokines such as IFN-γ, TNF-α, and IL-1β and is functionally decoupled from intracellular calcium levels due to its high-affinity interaction with calmodulin, thereby enabling sustained, high-output NO production (148). Additionally, p53 negatively regulates NOS2 by repressing its basal and cytokine-induced expression in response to elevated NO levels, forming a feedback loop to restrain NO production (149).

In malignant contexts, NOS isoforms exhibit distinct expression patterns across diverse cellular compartments of the tumor microenvironment, including neoplastic cells, infiltrating immune subsets, stromal fibroblasts, and vascular endothelium, collectively orchestrating the spatial and temporal regulation of nitric oxide bioavailability within the evolving tumor niche (140).

Among immune populations, TAMs constitute a principal source of NO. Classically activated M1-polarized TAMs robustly express NOS2 and generate micromolar levels of NO with cytotoxic and pro-inflammatory properties, promoting tumor cell lysis and antigen presentation (150, 151). However, under the influence of tumor-derived factors such as IL-10, TGF-β and hypoxia, TAMs frequently adopt an M2-like phenotype characterized by diminished NOS2 expression and increased arginase-1 (ARG1) activity (152). This phenotypic shift favors alternative L-arginine metabolism, suppresses T cell effector function and fosters tumor progression. Interestingly, emerging evidence supports the existence of TAMs subsets co-expressing NOS2 and ARG1, suggestive of a hybrid activation state with concurrent pro-inflammatory and immunosuppressive functions (153, 154).

MDSCs, which accumulate in response to chronic inflammation and oncogenic signaling, also exhibit high NOS2 expression, particularly within the monocytic subpopulation (155).

Tumor cells themselves are often competent NO producers via inducible NOS2 expression, particularly in response to hypoxia, inflammatory cytokines and oncogenic stimuli including Ras, Myc, and NF-κB (156, 157). High NOS2 expression has been reported in a wide range of human malignancies, including but not limited to melanoma, colorectal, breast, and prostate cancers (158–160). Autocrine and paracrine NO signaling in tumor cells facilitates immune evasion, angiogenesis, proliferation, and resistance to apoptosis (14, 160). Although CAFs do not typically express NOS isoforms under homeostatic conditions, they indirectly contribute to NO enrichment in the TME by secreting chemokines (e.g., CXCL12, CCL2) and cytokines (e.g., IL-6, TGF-β) that recruit and polarize NOS2-expressing myeloid cells (161, 162).

Endothelial cells of the tumor vasculature predominantly express NOS3, which is activated in response to shear stress, calcium influx, and angiogenic factors such as VEGF (163). NOS3-derived NO promotes vasodilation, increases vascular permeability, and supports neovascularization (164). In addition to its hemodynamic functions, NOS3 activity has been implicated in tumor metabolic adaptation through cGMP-mediated upregulation of peroxisome proliferator-activated receptor gamma coactivator 1-alpha (PGC-1α), driving mitochondrial biogenesis and sustaining bioenergetic demands under hypoxic stress (165).

### 4.2 Immunomodulatory roles of NO in the TME

NO is a key immunoregulatory molecule that profoundly influences immune cell interactions in the TME. Its impact is highly context-dependent, influenced by factors such as NO concentration, cellular source and spatial distribution within the TME (**Table 3**) (196, 197).

**Table 3:** NO–mediated regulation of the tumor microenvironment.

NO role	Cell types	Molecular mechanisms	Experimental models	References
Pro-tumoral	Cancer cells (melanoma, breast, colorectal, prostate, NSCLC, ovarian)	sGC/cGMP/PKG, MAPK, PKC → ↑ proliferation, ↓ caspases, ↑ BCL-2	In vitro tumor cell lines; in vivo xenografts	(139–142, 147)
	Cancer cells (CRC, breast, lung)	Inhibition of PHDs → HIF-1α stabilization under normoxia → ↑ VEGF, glycolysis	In vitro; in vivo	(143)
	Cancer cells (breast, melanoma)	S-nitrosylation of EGFR/Src → activation of Akt, c-Myc, β-catenin; inhibition of PP2A	In vitro; in vivo	(166)
	Cancer stem cells (NSCLC, glioma)	S-nitrosylation of Notch1 → UCHL1-mediated stabilization → CSC maintenance, radio-resistance	In vitro; in vivo	(167–169)
	Cancer cells (breast)	↑ VEGF-C → lymphangiogenesis and lymph-node metastasis	In vivo	(170)
	Cancer cells (ovarian sarcoma)	Enhanced migration and metastatic dissemination	In vivo	(171, 172)
	TAMs (M2-like) (breast, pancreatic, melanoma)	L-arginine depletion, IL-10/TGF-β and hypoxia→ T-cell suppression	In vivo	(152, 154)
	MDSCs (M-MDSCs) (melanoma, prostate, lung)	NO/ONOO^−^ → TCR nitration, IL-2 blockade, T-cell dysfunction	In vitro; in vivo; human samples	(155, 173, 174)
	Endothelial cells (breast, melanoma, CRC)	sGC/cGMP, PI3K/Akt → vasodilation, permeability, angiogenesis	In vitro (HUVECs); in vivo	(163, 164, 175, 176)
Anti-tumoral	TAMs (M1-like) (melanoma, fibrosarcoma, CRC)	DNA damage, mitochondrial inhibition → tumor cell lysis	In vitro; in vivo	(150, 151, 154)
	Macrophages, NK, endothelial cells (sarcoma, lymphoma)	Inhibition of aconitase & ribonucleotide reductase → ↓ DNA synthesis	In vitro; in vivo	(177, 178)
	Cancer cells (ovarian, lung, prostate)	ROS/RNS, ONOO^−^ → p53 activation, caspases, G2/M arrest	In vitro; in vivo	(142, 179–181)
	Cancer cells (CRC, breast)	Inhibition of NF-κB, Wnt/β-catenin, MAPK survival pathways	In vitro	(182, 183)
	Cancer cells (ovarian, lung)	Chemo- and radio-sensitization (cisplatin, TRAIL, RT)	In vitro; in vivo	(183–185)
	Activated fibroblasts (fibrosarcoma)	Stromal NO → tumor suppression, reduced metastasis	In vivo (NOS2^−^/^−^ mice)	(186–188)
	Endothelial cells (breast, lung)	Anti-angiogenic effects via ERK/PKC inhibition	In vitro; in vivo	(189, 190)
	Immune compartment (melanoma, ovarian)	Feedback inhibition of iNOS, ↓ ONOO^−^, restored T-cell immunity	In vivo	(191–195)
	Cancer cells (prostate)	EMT reversal → ↓ invasion and metastasis	In vitro	(171)
	Cancer cells (melanoma)	Reduced metastatic potential	In vivo	(172)

This table summarizes the context-dependent roles of nitric oxide (NO) within the tumor microenvironment, highlighting its pro- and anti-tumoral effects. For each cell population, the table reports the tumor types investigated, the main molecular mechanisms regulated by NO, the experimental models used (*in vitro* and/or *in vivo*), and the corresponding references. The data illustrate how the cellular source and microenvironmental context shape the impact of NO on tumor progression, immune regulation, and therapy response.

Originally, NO was recognized primarily for its essential role in the tumoricidal activity of TAMs (198). Specifically, activation of the NF-κB signaling pathway in pro-inflammatory (M1-like) macrophages induces expression of the *iNOS* gene, leading to robust NO production (199). The NO produced in this context supports anti-tumor immunity by exerting cytoprotective effects that enhance the survival of other key immune cells involved in the anti-tumor response, such as DCs and monocytes (196). However, more recent evidence from Drehmer et al. highlights the complexity of NO’s role in the TME (200). The study shows that at concentrations beyond those inhibiting cellular respiration, NO promotes the maintenance of a pro-inflammatory environment.

Macrophages exposed to high NO levels exhibit a dysfunctional phenotype which may lead to the persistence of inflammation while impairing adaptive immunity, ultimately supporting tumor development and progression (200). Tumor cells may exploit the immunosuppressive properties of NO to evade immune surveillance. For instance, Liu et al. showed that melanoma cells can suppress interferon responses in peripheral blood mononuclear cells (PBMCs) from healthy donors, and this suppression correlated negatively with NOS1 expression (201). Moreover, high NOS1 levels were associated with resistance to adoptive T cell transfer therapies in melanoma metastases (201). The ability of tumors to exploit NO functions has been also demonstrated by the observation that tumor-derived prostaglandin E2 (PGE2) induces nuclear accumulation of p50 NF-κB in M-MDSCs, diverting their response to IFNγ towards NO-mediated immunosuppression (202). Moreover, it has been shown that NO stabilizes pro-inflammatory M1 phenotypes by impairing oxidative phosphorylation, thereby preventing M2 repolarization (203, 204). Ultrasound-responsive NO-releasing nanoparticles further promoted M1 polarization and dendritic cell activation while depleting MDSCs (205). Furthermore, iNOS-expressing macrophages were essential for CD8+ T cell recruitment through the induction of endothelial adhesion molecules (e.g., VCAM-1) and Th1 chemokines (206).

Other myeloid populations are shaped by NO signaling in the TME. Notably, tumor-expressed *NOS2* recruits and activates MDSCs, partly via VEGF, as shown in melanoma models (207), while tumor-derived factors (GM-CSF, VEGF, IL-6) induce NOS2 in MDSCs through STAT3 and NF-κB, stabilizing their suppressive phenotype (208). PMN- and M-MDSC subsets employ NO-dependent mechanisms to inhibit anti-tumor immunity: NO nitrosylates tyrosine residues in TCR components, disrupts IL-2 signaling, and impairs antigen-specific T cell responses, often via peroxynitrite (ONOO^−^) formation (173, 174). Elevated nitrotyrosine in tumor-infiltrating lymphocytes correlates with T cell dysfunction, reversible by NOS2 or arginase inhibition. NO also affects T cell trafficking S-nitrosylating CCL2 to limit CD8^+^ T cell recruitment while allowing MDSCs accumulation (191). Notably, pharmacological NOS2 inhibition (209) or NO scavengers (e.g., carboxy-PTIO) reduces MDSC-mediated immunosuppression and restores CD8^+^ T cell infiltration and cytolytic activity in preclinical models (210).

Beyond its direct effects on T cells, MDSC-derived NO disrupts multiple immune functions by impairing DC-mediated antigen presentation to CD4^+^ T cells (192) and suppressing NK cell cytotoxicity through protein tyrosine nitration (211). In melanoma, NOS2 also promotes a protumoral IL-17–producing γδ T cell phenotype that recruits MDSCs and dampens γδ T cell cytotoxicity (212).

Despite this immunosuppressive role, exogenous NO donors can counterintuitively exert beneficial effects in specific contexts (139). GSNO reduced MDSC accumulation, restored T cell proliferation, and increased IFN-γ–producing CD4^+^ and CD8^+^ T cells in ovarian cancer models, slowing tumor growth (193). Low NO levels selectively promote Th1 polarization via cGMP-dependent IL-12R upregulation (194). Moreover, NO donors such as AT38 and NO-releasing aspirin suppress intratumoral iNOS activity, peroxynitrite formation, and CCL2 nitration, thereby enhancing T cell–based immunotherapies and vaccination efficacy (191, 192). These effects likely involve feedback inhibition of NOS expression and activity through S-nitrosylation and NF-κB suppression (195), indicating that exogenous NO can counteract immune dysfunction driven by excessive NOS2 signaling.

### 4.3 NO-mediated modulation of stromal and tumor cells within the TME

Like the other gasotransmitters, NO plays pivotal role in orchestrating the intricate network of the TME, including modulation of ECM, vasculature and the diverse array of immune cell populations. NO is a highly reactive molecule, especially toward the nucleic acids, and sustained exposure can lead to genotoxic stress and accumulation of mutations (213). Moreover, NO can modify cancer cell metabolism inducing the Warburg effect and influencing the response to chemotherapeutic agents (214, 215). NO can also promote glycolysis in ovarian cancer by interacting with PKM2, an isoenzyme of the glycolytic enzyme pyruvate kinase, and facilitate its nuclear translocation (216). During tumor progression, NO is engaged in regulating multiple biological processes, including angiogenesis, immune evasion and metastatic dissemination (15). The NO pro-tumorigenic impact on tumor growth has been extensively investigated in experimental models; while genetic overexpression of *NOS2* in cancer cells enhances proliferation, antisense suppression of *NOS2* attenuates tumor cell growth (149, 217). Nevertheless, a substantial body of evidence highlights the anti-proliferative role of NO; notably, NO produced by macrophages, Kupffer cells, NK and endothelial cells has been shown to induce cytostatic and cytotoxic effect in various tumor types, targeting key enzymes such as aconitase and ribonucleotide reductase (177, 178). These interactions lead to the inhibition of DNA synthesis and activation of cell death pathways, including salvage mechanisms (218). Accordingly, high and sustained concentrations of NO have been associated with the activation of pro-apoptotic cascades via mitochondrial pathways, upregulation of *wild-type* p53 onco-suppressor and induction of pro-apoptotic Bcl-2 family (142).

Interestingly, cytokine-activated fibroblasts within the TME can exert tumoricidal activity via *NOS2*-derived NO (186, 187), and stromal-derived NO has been shown to suppress fibrosarcoma progression and metastasis in *NOS2*^–/–^ mice (188). Mechanistically, NO induces apoptosis through a multitude of redox-sensitive and signaling-dependent pathways; it promotes oxidative stress by increasing ROS and depleting antioxidants like glutathione, thereby activating caspases and inducing mitochondrial damage (179). Moreover, DNA double-strand breaks, activation of JNK signaling and shifts in the Bax/Bcl-2 ratio toward apoptosis have been reported (180, 181, 197). Importantly, NO downregulates survival-promoting pathways such as NF-κB, Wnt/β-catenin and MAPK signaling, reinforcing its anti-tumoral potential (182, 183). NO also interferes with cell cycle progression, particularly arresting cells in the G2/M phase through modulation of cyclins and cyclin-dependent kinases (179). Its role as a chemosensitizer and radiosensitizer has been validated in several models; for instance, *NOS2* transfection enhances cisplatin-induced apoptosis, and NO amplifies TRAIL-mediated cytotoxicity via NF-κB inhibition (183). Radiosensitizing effects have been attributed to NO-mediated increases in tumor perfusion and activation of p53-dependent apoptosis (184, 185).

Nonetheless, the effect of NO is not universally tumor suppressive. Certain cancers, particularly those with p53 mutations, may exhibit resistance or even proliferative responses to NO exposure (149). Furthermore, NO can facilitate oncogenic signaling through S-nitrosylation of EGFR and Src, activating c-Myc, Akt, and β-catenin pathways in basal-like breast cancer while inhibiting tumor suppressors like PP2A (166). Moreover, in breast cancer, elevated NO correlates with increased VEGF-C expression and lymph node metastasis, highlighting its role in promoting angiogenesis and tumor dissemination (170).

Angiogenesis is tightly regulated by NO. In hypoxic tumor regions (<5% oxygen), stabilization of HIF-1α and HIF-2α induces pro-angiogenic factors (VEGF, FGF-2, IL-8, PDGF), a program further amplified by low NO levels, which activate MMPs (MMP-1, -9, -13) to remodel the ECM and facilitate endothelial invasion and sprouting (219). Consistently, NOS2 expression correlates with increased VEGF levels and microvascular density across multiple tumors (149). In *in vitro* investigations using human umbilical vein endothelial cells (HUVECs), basal NOS3 activity supports survival and proliferation via PKA/PI3K/Akt signaling (175), and VEGF-driven angiogenesis relies on Akt-dependent eNOS phosphorylation mediated by Hsp90 (176). In contrast, supraphysiological NO levels delivered by donors (e.g., DETA-NONOate, SNP) inhibit endothelial proliferation and tube formation through suppression of MAPK/ERK and PKC pathways, underscoring the concentration-dependent dual role of NO in tumor angiogenesis (189, 190).

Additionally, NO intersects with key pathways that promote tumor invasion and metastasis (220). Both iNOS and eNOS support cancer cell migration and invasion in breast and colon cancers via sGC activation and MAPK signaling (221). NO also contributes to epithelial–mesenchymal transition (EMT): in breast cancer cells, exogenous NO induces EMT features, including E-cadherin downregulation and vimentin upregulation (222, 223). Conversely, sustained high NO levels delivered by DETA-NONOate can reverse EMT and suppress invasiveness in metastatic prostate cancer, underscoring a concentration- and context-dependent role (171). Consistently, NOS2 deficiency enhances metastasis in ovarian sarcoma but reduces it in melanoma, highlighting tumor-type specificity (172). Beyond invasion, NO supports cancer stem cell (CSC) maintenance and therapy resistance. In NSCLC, NO-mediated S-nitrosylation stabilizes Notch1 via UCHL1, preserving stemness and radioresistance, while NOS inhibition reduces CSC traits (167). Furthermore, tumor-intrinsic NOS2 also correlates with CSC marker expression, neurosphere formation, and SOX-2 upregulation in glioma (168). Accordingly, NOS2 ablation in KRAS-driven lung cancer delays tumor onset and reduces metastasis (169), and NOS2-deficient pancreatic tumors display reduced invasiveness and EMT marker expression, further linking NO signaling to metastatic progression (224).

## 5 Crosstalk between gasotransmitter pathways

Emerging evidence indicates that gasotransmitter signaling within the TME operates as a highly integrated and adaptive network rather than as a set of independent linear pathways, with extensive crosstalk among CO/HO-1, H_2_S/CBS–CSE–3-MST and NO/NOS systems orchestrating tumor–stroma–immune interactions (14). The interplay between NO and H_2_S is bidirectional and highly context dependent. NO increases CSE expression while inhibiting CBS, resulting in differential modulation of H_2_S levels across tumor and stromal compartments (225, 226). In turn, H_2_S enhances eNOS expression and activity via intracellular Ca²^+^ release, Akt-mediated phosphorylation, and S-sulfhydration, which stabilizes eNOS dimers and sustains NO production (227). H_2_S also mitigates oxidative stress and scavenges peroxynitrite, thereby preserving NO bioavailability within the oxidative TME (228–230). H_2_S can both upregulate and suppress iNOS-derived NO depending on inflammatory cues and NF-κB activation, also via HO-1 expression, highlighting its dual regulatory potential in macrophages (231, 232). Beyond synthesis, H_2_S augments downstream NO signaling through phosphodiesterase type 5 (PDE5A) inhibition and redox sensitization of sGC, prolonging cGMP accumulation and amplifying NO-dependent vascular and immunomodulatory effects (233). Moreover, NO and H_2_S react chemically to generate bioactive intermediates, including nitrosothiols, HSNO, nitroxyl and polysulfides, which can exert enhanced or distinct actions relative to the parent gasotransmitters (234, 235).

CO contributes a further layer of regulation within the TME. It modulates eNOS and iNOS activity in a dose- and tissue-dependent manner, activating eNOS through Ca²^+^–IP_3_–Akt signaling and protecting against inflammatory downregulation, while inhibiting iNOS via NF-κB suppression and PPAR-γ activation (236, 237). CO can elevate NO levels by competing for intracellular binding sites, but at higher concentrations, it may inhibit NO release, illustrating concentration-dependent duality (238). Conversely, NO and peroxynitrite upregulate HO-1 expression through mRNA stabilization, establishing a cytoprotective feedback mechanism in tumor-associated endothelial and immune cells (239). CO also directly inhibits CBS while enhancing CSE expression, indirectly promoting H_2_S production, whereas H_2_S modulates CO availability via HO-1 expression; such reciprocal regulation has been observed in models of gastric injury and chronic kidney disease, where both gasotransmitters exert interdependent cytoprotective effects (240–242).

Importantly, NO, H_2_S, and CO also compete for hemoglobin binding, forming nitrosyl hemoglobin, green sulfhemoglobin and carboxyhemoglobin, which may influence vascular tone, oxygen delivery and local gasotransmitter activity within TME (243–245). Functionally, these intertwined pathways govern critical aspects of tumor biology: CO promotes M2-like polarization of TAMs, H_2_S modulates T-cell activation and myeloid metabolism via persulfidation of NF-κB, STAT3 and metabolic enzymes and NO/H_2_S signaling coordinates endothelial barrier function and angiogenesis (231, 232). Perturbation of one gasotransmitter pathway often triggers compensatory responses in others, highlighting the necessity of considering these molecules as an integrated network when designing therapeutic strategies to modulate immune evasion, stromal remodeling and angiogenesis in cancer (14, 246).

## 6 Preclinical perspectives and therapeutic potential of gasotransmitter modulation

The endogenously synthesized NO, CO and H_2_S have garnered significant attention as multifaceted modulators of oncogenic processes and as promising molecular entities for therapeutic exploitation (13, 247). While historically appreciated for their canonical roles in the regulation of vascular homeostasis, synaptic transmission and immunological responses, these gaseous mediators have since been implicated in the orchestration of numerous cancer hallmarks, exhibiting pleiotropic effects that are often highly dependent on their local concentration, cellular context and temporal dynamics (14). Preclinical data attests the anti-tumorigenic potential of NO, CO, and H_2_S, demonstrating their capacity to suppress neoplastic proliferation, induce cell cycle arrest and programmed cell death, inhibit EMT and constrain invasive and metastatic behavior. Furthermore, these molecules have been shown to exert profound effects on the TME, enhancing anti-tumor immune surveillance and modulating stromal-immune interactions in favor of tumor suppression (19). Paradoxically, these same gasotransmitters may, under conditions of aberrant expression or dysregulated signaling, contribute to tumor progression by fostering prosurvival signaling cascades, promoting angiogenesis and attenuating anti-neoplastic immune responses. This intrinsic duality reflects the complex and context-sensitive nature of gasotransmitter biology and underscores the imperative for exquisitely targeted modulation strategies to harness their therapeutic potential without inadvertently promoting malignancy (19, 248).

Gasotransmitter signaling in cancer is both spatially and temporally compartmentalized, reflecting the marked cellular, metabolic and vascular heterogeneity of the TME. NO, CO and H_2_S are produced in discrete tumor niches, including hypoxic cores, invasive fronts, perivascular regions, stromal fibroblasts and immune infiltrates, resulting in steep, micrometer-scale concentration gradients (19, 139, 249). In hypoxic and nutrient-limited regions, CBS–derived H_2_S sustains mitochondrial respiration and redox homeostasis, whereas CSE predominates in stromal and endothelial compartments (27, 34); similarly, HO-1–derived CO accumulates in perivascular stromal cells and TAMs, promoting localized immunosuppressive and pro-angiogenic microdomains (94, 250). NO signaling occurs in highly dynamic, transient pulses generated by iNOS and eNOS rather than sustained exposure (251). These spatial gradients and temporal dynamics critically determine biological outcomes, as neighboring cells may experience gas concentrations ranging from nanomolar pro-survival signals to cytotoxic micromolar bursts, providing a mechanistic basis for the context-dependent pro- and anti-tumor effects of gasotransmitters (14, 82).

Recent advances in chemical biology and imaging technologies now permit direct visualization of gasotransmitters in living systems with high spatial and temporal resolution, providing new insights into their roles in tumor progression. Fluorescent and bioluminescent probes reveal elevated H_2_S at invasive and hypoxic tumor regions, correlating with CBS expression, while genetically encoded sensors distinguish cytosolic from mitochondrial H_2_S pools (252–254). For NO, metal-based fluorescent probes, electron paramagnetic resonance (EPR) spin trapping, and genetically encoded NO-sensitive reporters have captured rapid, compartmentalized NO bursts in immune and endothelial cells of the TME (255–257). Although CO detection has historically been challenging, newly developed palladium- and ruthenium-based fluorescent probes and heme protein–derived biosensors now permit real-time monitoring of HO-1–dependent CO generation *in vivo*, yet their application in whole-animal models remains limited due to challenges in delivery, sensitivity and tissue penetration (258–260).

### 6.1 Immunomodulatory roles and therapeutic outcomes of H_2_S modulation

*In vitro* studies using genetic knockdown (siRNA/shRNA) and pharmacologic inhibitors such as aminooxy acetic acid (AOAA) demonstrate that CBS-derived H_2_S promotes tumor cell proliferation, migration, and invasion (27, 28). CBS downregulation or inhibition suppresses tumor bioenergetics by impairing mitochondrial electron transport, oxidative phosphorylation and glycolysis (27). In ovarian cancer models, CBS inhibition also reduces intracellular antioxidant glutathione and triggers apoptosis through modulation of NF-κB and p53 pathways (28). Notably, CBS silencing increases intracellular ROS, potentially sensitizing tumor cells to immune-mediated cytotoxicity, as seen in breast cancer models (261). *In vivo*, stable CBS knockdown in colon and ovarian cancer xenografts leads to 40–50% reduction in tumor growth, diminished tumor nodule size and number and inhibition of peritumoral angiogenesis (27, 28). AOAA treatment recapitulates these effects with superior efficacy, likely due to off-target actions beyond CBS inhibition (27, 262). Importantly, CBS inhibition also sensitizes tumor cells to chemotherapy (263). These findings collectively indicate that CBS-derived H_2_S creates a supportive microenvironment for tumor progression. However, the role of CBS is tumor-type dependent, as exemplified by glioma models where CBS silencing paradoxically enhances tumor growth (264). Similarly, silencing CSE inhibits tumor growth in colon cancer (265) but not in melanoma (266), highlighting enzyme- and context-dependent functions. Interestingly, Nafea et al. recently demonstrated that inhibiting H_2_S production through the microRNA miR-939-5p-mediated suppression of CBS and CSE effectively reduces the growth and progression of triple-negative breast cancer, underscoring the antitumor potential of targeting H_2_S synthesis (29). AOAA remains the most potent CBS inhibitor identified, exhibiting an IC_50_ of 3–10 μM against human recombinant CBS, though it lacks selectivity due to inhibition of other transaminases (262). Preclinical studies in tumor-bearing mice indicate that AOAA prodrugs demonstrate enhanced cellular uptake and superior anticancer efficacy compared to the parent compound (267).

Interestingly, a study employing both genetic (CBS^+^/^−^ mice) and pharmacological inhibition of CBS in colorectal cancer-bearing mice reported a significant reduction in CD4^+^CD25^+^Foxp3^+^ regulatory T cell populations across both spleen and tumor tissues, coupled with a notable increase in the CD8^+^ T−cell/Treg ratio (51). This immunological shift was associated with enhanced responses to anti−PD−L1 and anti−CTLA−4 therapy, demonstrating the pivotal role of H_2_S in maintaining an immunosuppressive tumor microenvironment (51).

Endogenously produced H_2_S acts as a metabolic integrator that supports cancer cell survival and proliferation (24); it also facilitates DNA repair fidelity by persulfidating MEK1 and activating PARP-1, helping tumor cells resist genotoxic chemotherapy (268).

Pharmacological delivery of H_2_S donors such as the slow-release GYY4137 or hybrid molecules like HA-ADT induces cytostatic and cytotoxic effects (269). These agents reduce proliferation, cause G2/M cell cycle arrest, impair mitochondrial function, and activate intrinsic apoptosis pathways, often via caspase-9 (269). For example, GYY4137 suppresses STAT3 phosphorylation and downregulates cyclin D1 and VEGF in hepatocellular carcinoma models, inhibiting tumor growth and angiogenesis (270). Preclinical evidence shows that exogenous H_2_S donors can potentiate standard cancer treatments. GYY4137 synergizes with chemotherapeutics like paclitaxel in colorectal cancer by enhancing apoptosis and lowering drug IC_50_ values without harming normal cells (271). Additionally, novel H_2_S-releasing hybrid compounds targeting tumor-enriched enzymes, including carbonic anhydrase, have demonstrated potent anti-cancer effects under hypoxic conditions, inducing cell cycle arrest and apoptosis in breast, colon, and lung cancer lines (272). Moreover, conjugation of H_2_S-releasing moieties with established drugs, such as in NOSH-aspirin hybrids (donating both nitric oxide and H_2_S), yields compounds with superior anti-proliferative and anti-metastatic properties. These hybrids target multiple signaling pathways including COX-2 inhibition, oxidative stress modulation, and mitochondrial disruption, providing a multifaceted therapeutic approach (273).

### 6.2 Immunomodulatory roles and therapeutic outcomes of HO-1/CO modulation

Given its complex roles, the HO-1/CO axis presents both therapeutic challenges and opportunities in oncology. Preclinical studies utilizing HO-1 knockout mice or pharmacological HO-1 inhibitors, such as zinc protoporphyrin (ZnPP) or tin protoporphyrin (SnPP) and imidazole-based compounds (e.g. OB-24) have demonstrated impaired tumor growth, reduced angiogenesis and restoration of antitumor immune responses, corroborating the pro-tumoral role of HO-1/CO under physiological levels (139, 274). Elevated HO-1 expression and CO production have been correlated with resistance to chemotherapy and radiotherapy, poor prognosis and metastatic potential in several cancers (114). Consequently, inhibition of HO-1 or reduction of CO levels has been explored as a strategy to enhance the efficacy of standard cancer treatments (275).

In the context of chemotherapy, CO impairs drug-induced apoptosis by preserving mitochondrial membrane potential, reducing cytochrome c release and inhibiting caspase activation. Preclinical models have shown that siRNA-mediated knockdown of HO-1 sensitizes non-small cell lung cancer (A549) and triple-negative breast cancer (MDA-MB-231) cells to cisplatin and doxorubicin, respectively, through increased ROS accumulation and caspase-3/9 activation (276, 277). *In vivo*, treatment with HO-1 inhibitors like ZnPPIX or tin mesoporphyrin (SnMP), in combination with chemotherapy, leads to enhanced tumor regression and prolonged survival (98). Specifically, ZnPPIX treatment sensitizes neuroblastoma cells to glutathione depletion and etoposide treatment as well as to bortezomib (278). Moreover, in BRAFV600-mutated melanoma cells, SnPPIX enhances cell death induced by vemurafenib (PLX4032) (279). Accordingly, OB-24 suppresses proliferation of advanced prostate cancer cells *in vitro* and reduces tumor growth and metastasis to lymph nodes and lungs *in vivo*, exhibiting strong synergy with taxol (280).

HO-1/CO signaling also contributes to therapy resistance via stabilization of HIF-1α and promotion of tumor hypoxia, both of which sustain angiogenesis and metabolic reprogramming. Disrupting this axis reverses hypoxic adaptation and sensitizes tumors to chemotherapeutic agents (19). Moreover, thermally triggered CO-releasing systems have been shown to reverse chemotherapy resistance in doxorubicin-resistant MCF-7/ADR tumors by inducing mitochondrial exhaustion, leading to ATP depletion, inhibition of ATP-dependent doxorubicin efflux and promoting apoptosis via caspase-3 activation (134). Inhibition of HO-1 was also reported to enhance the therapeutic efficacy of nab-paclitaxel combined with gemcitabine in pancreatic ductal adenocarcinoma by increasing tumor cell sensitivity to treatment (281). Additionally, HO-1 inhibition alters the TME, reducing pro-tumorigenic factors and promoting anti-tumor immune responses (281). Furthermore, in an aggressive spontaneous murine model of breast cancer (*MMTV-PyMT*), treatment with 5-fluorouracil (5-FU) has been shown to synergize with pharmacological HO-1 inhibition using SnMP, effectively reversing immunosuppression and promoting CD8+ T cell-mediated tumor growth control (98). Similarly, HO-1 is implicated in resistance to radiotherapy. Ionizing radiation upregulates HO-1 expression in several tumor models, including pancreatic and colorectal cancer (128). Silencing or inhibiting HO-1 increases radiation-induced DNA damage and enhances ROS-mediated cytotoxicity, as evidenced by increased γH2AX foci and reduced clonogenic survival (282). These findings support the use of HO-1 inhibitors as radiosensitizers in otherwise resistant tumors. Beyond its cytoprotective role, CO has profound effects on the immune landscape of the TME. In murine melanoma (B16-F10) and colorectal carcinoma (MC38) models, HO-1 inhibition or myeloid-specific HO-1 deletion shifts macrophages to a pro-inflammatory M1 phenotype, enhances IL-12 and TNF-α secretion and supports CTL and NK cell activation (94, 103). Notably, combining HO-1 blockade with immune checkpoint inhibitors (anti-PD-1 or anti-CTLA-4) results in synergistic antitumor effects, with improved CD8^+^/Treg ratios, decreased expression of exhaustion markers (e.g., PD-1, LAG-3), and enhanced tumor regression (275). In murine models of B16-F0 melanoma, the combination of OB-24 with anti-PD-1 therapy significantly enhances tumor regression compared to monotherapy. This effect is attributed to HO-1 inhibition, which increases tumor susceptibility to immune-mediated killing and prevents CD4+ and CD8+ TIL evasion (283). In preclinical model of melanoma and fibrosarcoma, pharmacological inhibition of HO-1 using ZnPPIX or myeloid-specific HO-1 deletion has been shown to prevent metastasis formation and enhance the effectiveness of anti-PD-1 immunotherapy (94). These immunomodulatory effects are further supported in HO-1 knockout mouse models, which exhibit stronger responses to immunotherapy and reduced tumor burden (17). Additionally, HO-1 targeting sensitizes hematological malignancies such as chronic myeloid leukemia to tyrosine kinase inhibitors and proteasome inhibitors (284).

Conversely, preclinical studies have also demonstrated that controlled CO delivery induces tumor cell apoptosis, enhances immune-mediated tumor clearance and improves responses to immunotherapy (285). Exogenous CO administration has been reported to induce immunogenic cell death in both *in vitro* and *in vivo* 4T1 breast cancer models, thereby enhancing anti-tumor immunity through dendritic cell maturation, increased CD4+ and CD8+ T cell infiltration, an improved CD8+/Treg cell ratio, and potentiation of anti-PD-L1 checkpoint therapy efficacy (286). Thus, this dualistic nature underscores the necessity for precise regulation of CO release from CORMs to avoid inadvertently fostering pro-tumoral processes within the TME (287).

### 6.3 Immunomodulatory roles and therapeutic outcomes of NO modulation

NO plays a diverse role in various human cancers and a full comprehension of its actions is fundamental for devising novel antitumoral therapies. NO has a role in breast carcinoma development since a higher NOS activity has been found in invasive breast tumors (288) and estrogen stimulates eNOS release in breast tissue, which may promote the progression of metaplastic epithelium into carcinoma (289). Increased levels of NO have also been found in cervical cancer, lung cancer, gastric cancer, brain tumors and other types of tumors, where it promotes tumor growth and shows mutagenic and carcinogenic activities (15). As previously stated, NO possesses tumoricidal effect when present in high concentrations. NO derived from macrophages, kupfer cells, NK and endothelial cells participates in tumor suppressor activities (290). These findings paved the way for several new anticancer therapies based around the manipulation of *in vivo* NO production.

Exogenous NO donors, including organic nitrates, diazeniumdiolates and metal–nitrosyl complexes, circumvent the dependence on enzymatic NOS activity by delivering NO or NO^+/-^ species directly to target tissues through distinct release kinetics and tissue distribution profiles (291). Their principal anticancer utility arises from the ability to normalize aberrant tumor perfusion, alleviate hypoxia-driven resistance and modulate redox-sensitive survival pathways (292). To optimize the release of NO, these donors have been incorporated with biopolymers or nanoparticles such as PEGylated polymer micelles (293) or hydrogel/glass hybrid nanoparticles (294). Quantum Dots can also be linked to NO-donor molecules and lead to effective treatment of large tumors via photodynamic therapy (295). Nitro-glycerine, a well-known NO donor, when administered as a chemo-sensitizing agent can act as a safe and affordable alternative for the management of resistant or metastatic tumors (296). In poorly vascularized tumors, NO donors, such as glyceryl trinitrate (GTN), can partially reverse therapeutic resistance by enhancing intratumoral blood flow and thereby improving oxygenation and chemotherapeutic delivery, counteracting the effects of regional hypoxia that stabilize HIF-1α and activate genes involved in angiogenesis, glycolysis and anti-apoptotic signaling (292). In a randomized phase II clinical trial, GTN significantly improved progression-free survival in patients with advanced non-squamous NSCLC when combined with cisplatin-based chemotherapy (297). Parallel clinical observations have demonstrated its therapeutic potential in hepatocellular (298), colorectal (299) and prostate cancer (300). Among the most extensively studied NO donors in preclinical settings are diazeniumdiolates (NONOates), which spontaneously release NO under physiological pH. DETA/NO has shown the ability to overcome chemoresistance to multiple agents, including 5-fluorouracil, cisplatin, doxorubicin and fludarabine, by enhancing drug-induced apoptosis via mitochondrial depolarization and caspase cascade activation (301, 302). Furthermore, the S-nitrosothiol GSNO has been demonstrated to reprogram TAMs, shifting the M2 pro-tumoral phenotype towards an M1 cytotoxic state, while concomitantly downregulating VEGF, AR, and AR-V7 expression in castration-resistant prostate cancer models (303). Intriguingly, by enhancing tumor perfusion, NO may also improve the delivery and efficacy of cytotoxic agents. Ji et al. demonstrated that NO-releasing and oxygen-delivering nanoparticles, activated by ultrasound, accumulated more efficiently in tumors with improved perfusion, thereby boosting the efficacy of sonodynamic therapy and antitumor immunity (205).

NO appears also to modulate several key pathways that influence the efficacy of immune checkpoint inhibitors (ICIs). The expression of PD-L1 on tumor cells is, in part, transcriptionally regulated by HIF-1α (304); NO donors have been shown to downregulate HIF-1α and its downstream targets, thereby reducing PD-L1 expression and enhancing T-cell–mediated tumor cytotoxicity (304, 305). Interestingly, in murine models of CT26 colon carcinoma, intratumoral delivery of ultra-high concentration gaseous NO (25,000–100,000 ppm, 10 s exposure) substantially upregulated PD-L1 expression on tumor cells *in vitro* and, when combined with anti–PD-1 therapy, achieved complete tumor regression in approximately 53% of animals and significantly enhanced CD8^+^ T-cell infiltration, M1 macrophage polarization, along with systemic immunologic memory and minimal observed toxicity (306). Biomaterial-enabled NO delivery systems, such as copper-laden, thermosensitive hydrogels co-loaded with NO donor and anti–PD-L1 antibodies, have also demonstrated potent antitumor efficacy in 4T1 breast cancer models (307). Conversely, NOS inhibition combined with PD-1 blockade has shown efficacy in humanized models of triple-negative breast cancer (TNBC); in these models L-NMMA, a non-selective NOS inhibitor, has been used in combination with pembrolizumab, a widely used PD-1 inhibitor. The combined molecules induced tumor regression in 66% of patient-derived xenografts—versus 40% with pembrolizumab alone—and NOS inhibition upregulated PD-L1 expression in TNBC cell lines, suggesting a context-dependent strategy of NO modulation (308). These divergent approaches reflect the nuanced, context-dependent effects of NO on tumor immunity and the critical need for patient-specific stratification strategies.

## 7 From bench to bedside: therapeutic application of gasotransmitters in cancer

### 7.1 Translational challenges for gas-based cancer immunotherapy

The clinical translation of gasotransmitters-based strategies in cancer immunotherapy is fundamentally limited by their unique pharmacokinetics and the highly context-dependent nature of their biological effects (309, 310). NO donors are rapidly inactivated via scavenging by hemoglobin and ROS, resulting in a short half-life and poor systemic bioavailability, whereas free CO and H_2_S cannot be administered systemically at therapeutic doses without causing substantial toxicity. A critical limitation therefore remains the lack of precise spatiotemporal control over gas delivery. To address these limitations, tumor-targeted strategies have emerged, including enzyme-activated prodrugs that exploit tumor-associated expression of iNOS, HO-1 or CBS, as well as stimuli-responsive nanoparticles releasing gas in response to hypoxia, acidic pH,or elevated glutathione (309, 311–313). Local delivery platforms, such as injectable hydrogels or implantable depots further improve specificity and prolong intratumoral exposure while minimizing systemic off-target effects (314).

Each gasotransmitter presents distinct therapeutic challenges. In particular, NO exhibits a narrow therapeutic window due to its short half-life and dual immunomodulatory and cytotoxic properties, necessitating careful dosing to exploit vasculature normalization and M1 macrophage polarization without inducing immunosuppression (315, 316). To overcome unfavorable pharmacokinetics and off-target effects, NO has been conjugated to NSAIDs, chemotherapeutics like doxorubicin and even non-traditional agents such as lopinavir, enhancing cytotoxicity and intratumoral accumulation (317, 318). However, these hybrid molecules often lack tumor specificity and remain limited by systemic toxicity. More refined targeting strategies, including antibody–drug conjugates incorporating NO donors or PDE-inhibitor, as well as metal–NO complexes, have shown enhanced cytotoxicity and selectivity in preclinical studies (319). In parallel, stimuli-responsive systems (e.g. light-activated NO–doxorubicin conjugates) and nanoformulations have further improved stability and spatial control (320). Yet, despite encouraging preclinical data, robust *in vivo* validation remains limited and clinical translation remains a challenge.

CO exerts anti-inflammatory and cytoprotective effects that, if constitutively elevated, can suppress host antitumor immunity; in contrast, selective HO-1 inhibition has been shown to alleviate myeloid-mediated immunosuppression and enhances checkpoint blockade efficacy (103, 106). Similarly, H_2_S regulates redox and metabolic signaling via protein persulfidation, and its overproduction has been implicated in T-cell exhaustion and immune evasion (21); in this context, slow-releasing donors or enzyme-targeted inhibitors can restore T-cell function and sensitize tumors to immunotherapy (321).

Importantly, tumor heterogeneity requires tailored gas-based interventions. Oxygen-releasing systems are most effective in hypoxic, immune-excluded tumors, while NO- or H_2_S-releasing depots may preferentially benefit stroma-rich or macrophage-dominated microenvironments (322, 323). Conversely, CO-based strategy may be exploited in post-operative or chronic inflammatory contexts due to its cytoprotective and anti-inflammatory properties (19). Biomarker-driven stratification, based on hypoxia signatures, myeloid phenotypes or redox enzyme expression, can therefore guide the selection of gas modality, dosing and delivery format for specific tumor niches. However, translational advances in cancer immunotherapy will require not only proof of local biocompatibility and sustained gas retention, but also the achievement of robust and clinically relevant immunological endpoints.

Given these constraints, gasotransmitter modulation is unlikely to achieve durable antitumor effects as a monotherapy. Instead, their greatest translational potential lies in rational combination with established immunotherapies (309). For example, HO-1 inhibitors such as tin mesoporphyrin (SnMP) have been shown to enhance T-cell infiltration and reverse myeloid-mediated immunosuppression when combined with anti–PD-1 therapy in preclinical models of advanced solid tumors, with early data suggesting improved immune activation in tumors with high HO−1 expression (98). Similarly, local NO delivery can normalize tumor vasculature and potentiate checkpoint blockade (324), while H_2_S-targeted strategies may enhance adoptive T-cell therapies or cancer vaccines by improving immune cell metabolic fitness and reducing exhaustion (51). These combinations leverage the capacity of gasotransmitters to remodel the TME, overcome hypoxia-driven resistance, and enhance antigen presentation, rather than relying solely on direct cytotoxicity.

Despite this progress, critical gaps remain, including optimization of dose scheduling, identification of predictive biomarkers (e.g., enzyme expression, hypoxia, redox status) and improved tumor-specific delivery platform to minimize systemic toxicity. Future strategies may integrate multifunctional nanocarriers, enzyme-activated prodrugs or spatially structured implants, complemented by advanced imaging technologies to guide personalized dosing and real-time monitoring of intratumoral gas levels (312).

In summary, gasotransmitter-based approaches are best viewed as precision adjuvants that synergize with established immunotherapies. By overcoming pharmacokinetic limitations, advancing tumor-targeted delivery systems and rationally designing combination regimens, controlled modulation of NO, CO and H_2_S holds promises to remodel the TME, strengthening immune effector function, and converting immunologically “cold” tumors into responsive disease, thereby narrowing the gap between preclinical efficacy and clinical translation.

### 7.2 Clinical translation of gas-based therapies in cancer immunotherapy

Gas-based therapies are increasingly being investigated for their potential to modulate the TME and enhance antitumor immunity through vascular normalization, immune cell infiltration and metabolic reprogramming (see **Table 4** for an overview of ongoing clinical trials). Among gasotransmitters, NO remains the most extensively studied. Intratumoral ultra-high concentration of NO is currently under evaluation in relapsed or refractory solid tumors (NCT05351502), while transdermal glyceryl trinitrate (GTN) delayed progression in recurrent prostate cancer (NCT01704274) (300), and a nitroglycerin patch combined with vinorelbine/cisplatin showed encouraging Phase II results in NSCLC, although it failed to demonstrate efficacy in the Phase III NVALT12 trial (297). iNOS inhibition with NG−monomethyl−L−arginine (L−NMMA) in combination with checkpoint inhibitors, including pembrolizumab and durvalumab (NCT03236935, NCT04095689), is being evaluated to mitigate NOS-driven immunosuppressive pathways and enhance antitumor immunity (325).

**Table 4:** Current clinical trials of gasotransmitter-based therapies in oncology.

Gas therapy	Gas donor	Administration	Gas-releasing system	Combination therapy	Phase status	Condition	Clinical trial ID	Reference
H2S	Methimazole	Oral intake	Endogenous H2S synthesis	Chemotherapy	Phase II (recruiting)	Progressive grade 4 gliomas	NCT05607407	Unpublished
NO	Direct gas	Intratumoral injection	N/A	Single	Phase I (recruiting)	Primary, metastatic, relapsed/refractory, or surgically unresectable cutaneous and subcutaneous malignancies	NCT05351502	Unpublished
NO	Glyceryl Trinitrate (GTN)	Local administration	Transdermal patch	Single	Phase II (completed)	Prostate-specific antigen (PSA) recurrence after primary therapy of prostate cancer	NCT01704274	(300)
NO	Nitroglycerin	Local administration	Transdermal patch	Chemotherapy (Vinorelbine and Cisplatin)	Phase II (completed)	Untreated stage IIIB/IV non-small cell lung cancer (NSCLC)	N/A	(297)

This table summarizes active or completed trials involving H_2_S and NO therapies, detailing the gas donor, administration route, delivery system, combination therapies, trial phase/status, target condition, clinical trial ID, and reference. H_2_S therapy (methimazole) is administered orally to enhance endogenous H_2_S synthesis and combined with chemotherapy in grade 4 glioma patients. NO therapies include direct gas via intratumoral injection or donors such as GTN and Nitroglycerin delivered through transdermal patches, applied as single agents or combined with chemotherapies (vinorelbine and cisplatin) in various cancers including cutaneous malignancies, prostate cancer and NSCLC. No clinical trials using CO therapy in oncology have been reported to date.

CO and H_2_S have undergone more limited clinical evaluation. Low-dose inhaled CO has demonstrated safety in non-oncologic contexts (326), whereas H_2_S modulation via methimazole (NCT05607407) is under investigation in recurrent gliomas to increase endogenous production, potentially enhancing chemotherapy through sulfhydration-mediated immunometabolic effects.

Oxygen-based strategies are also under evaluation. Hyperbaric oxygen combined with XELOX and anti–PD-1 for gastric cancer (NCT06742411) (327), as well as perfluorocarbon carriers in glioblastoma (NCT02189109; NCT03862430) (328), are under evaluation to alleviate tumor hypoxia and enhance the efficacy of immunochemotherapy. Molecular hydrogen (H_2_) has been reported to improve overall survival in lung cancer patients receiving nivolumab by restoring mitochondrial function and reversing CD8^+^ T-cell exhaustion (329), while ozone (O_3_) is being investigated preclinically for its immunomodulatory and oxidative effects (330).

The continued development of biocompatible and scalable delivery systems will be critical to fully realize the therapeutic potential of gas-based interventions in oncology.

## 8 Conclusion

**Figure 1:**
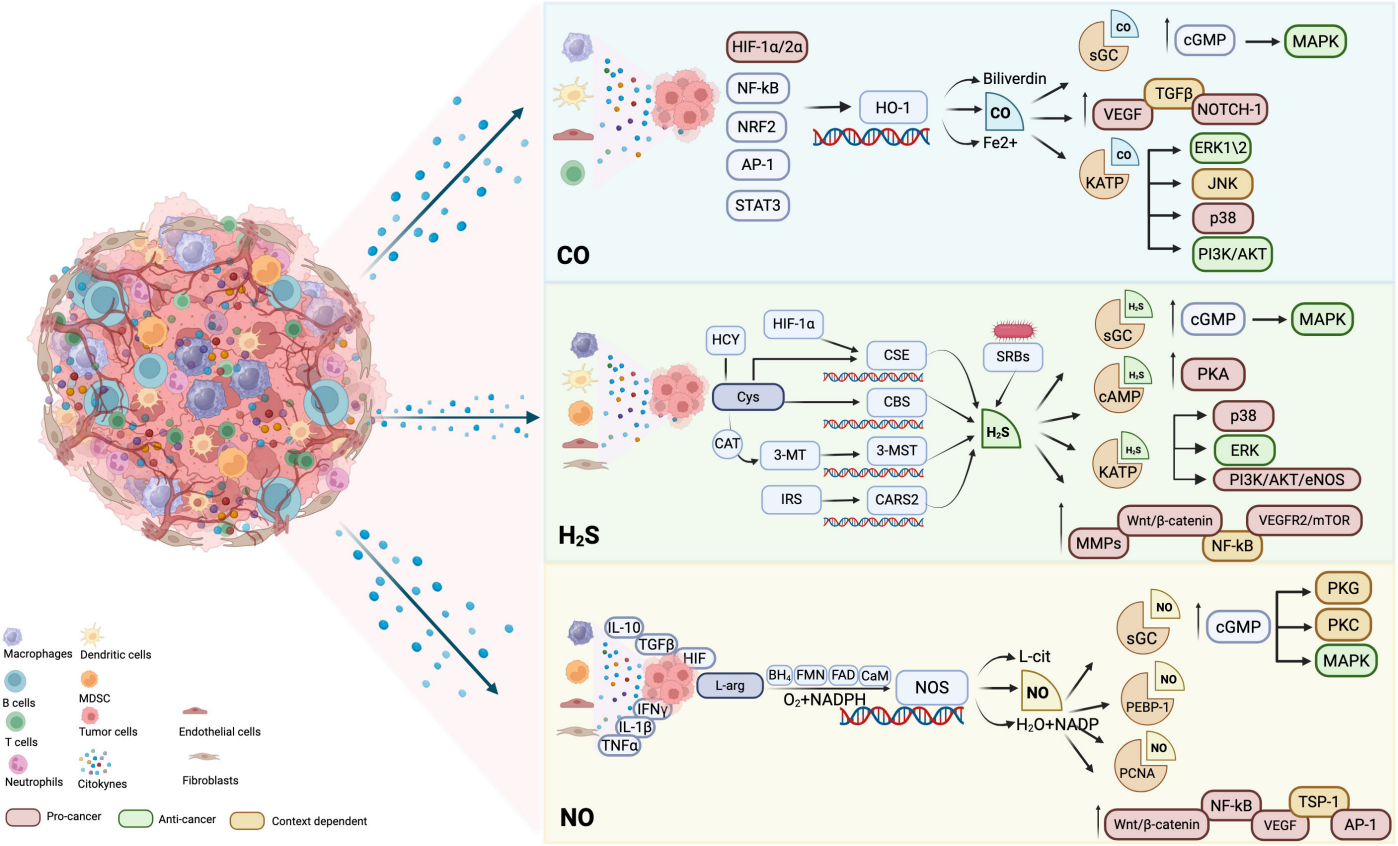
Immune-mediated signaling networks promoting gasotransmitters production in the TME. Gasotransmitters production is regulated by multiple signaling networks activated by different cell populations within the TME. As graphically represented in the figure, immune cells (including macrophages, dendritic cells, MDSCs and T cells), along with endothelial cells, fibroblasts and tumor cells release soluble factors that orchestrate several signaling cascades (i.e. HIF-1α/2α, NF-kB, NRF2, AP-1 and STAT3). These pathways drive the transcription of specific enzymes (HO-1; CSE, CBS, 3-MST, CARS2 and NOS) in immune, stromal and tumor cells ultimately resulting in gasotransmitters production (CO, H_2_S and NO. Depending on their concentrations and spatial localization within the TME, these intracellular mediators can exert either anti-inflammatory or pro-inflammatory effects, leading to context-dependent (yellow boxes) outcomes that may be either beneficial (anti-cancer effects-green boxes) or detrimental (pro-cancer effects- red boxes). (MDSC, Myeloid-Derived Suppressor Cells; HIF-1α/2α, Hypoxia-Inducible Factor 1-alpha/2-alpha; NF-κB, Nuclear Factor kappa-light-chain-enhancer of activated B cells; NRF2, Nuclear Factor Erythroid 2–Related Factor 2; AP-1, Activator Protein 1; STAT3, Signal Transducer and Activator of Transcription 3; HO-1, Heme Oxygenase-1; CO, Carbon Monoxide; sGC, Soluble Guanylate Cyclase; cGMP, Cyclic Guanosine Monophosphate; MAPK, Mitogen-Activated Protein Kinase; VEGF, Vascular Endothelial Growth Factor; TGF-β, Transforming Growth Factor β; NOTCH-1, Notch receptor 1; KATP, ATP-sensitive potassium channel; ERK1/2, Extracellular Signal-Regulated Kinase 1/2; JNK, c-Jun N-terminal Kinase; PI3K/AKT, Phosphoinositide 3-Kinase/Protein Kinase B; HCY, Homocysteine; Cys, Cysteine; CAT, Cysteine Aminotransferases; CSE, Cystathionine Gamma-Lyase; CBS, Cystathionine Beta-Synthase; 3-MT, 3-Mercaptopyruvate; 3-MST, 3-Mercaptopyruvate Sulfurtransferase; IRS, Insulin Receptor Substrate; CARS2, Cysteinyl-tRNA Synthetase 2; H_2_S, Hydrogen Sulfide; SRBs, Sulfate Reducing Bacyteria; cAMP, cycline Adenosine Monophosphate; PKA, Protein Kinase A; MMPs, Metalloproteinases; VEGFR2, Vascular Endothelial Growth Factor Receptor 2; L-arg, L-Arginine; BH_4_, Tetrahydrobiopterin; FMN, Flavin Mononucleotide; FAD, Flavin Adenine Dinucleotide; CaM, Calmodulin; NOS, Nitric Oxide Synthase; O_2_, Oxygen; NADPH, Nicotinamide Adenine Dinucleotide Phosphate; NO, Nitric Oxide; L-cit, L-Citrulline; PEBP-1, Phosphatidylethanolamine-Binding Protein 1; PCNA, Proliferating Cell Nuclear Antigen; PKG, Protein Kinase G; PKC, Protein Kinase C) Created by BioRender.

As the molecular mechanisms and context-specific signaling networks mediated by NO, CO and H_2_S are increasingly elucidated, their selective modulation is emerging as a promising strategy for adjunctive cancer therapy (331). Nonetheless, the clinical translation of these insights remains hampered by significant hurdles. The most compelling opportunities lies in targeting their immunomodulatory functions in the TME, where MDSCs, TAMs and other immune populations play pivotal roles in promoting immune evasion and tumor progression. Notably, several immune cell populations within the TME contribute to the endogenous production of these gasotransmitters (**Figure 1**), which often converge on overlapping signaling pathways, characterized by extensive crosstalk, compensatory mechanisms and high degree of redundancy (14). While such complexity reflects the sophistication of endogenous regulatory systems, it also complicates therapeutic interventions, as perturbation of a single pathway can trigger unintended dysregulation of parallel circuits, yielding unpredictable and potentially deleterious systemic effects.

Pharmacological approaches are further limited by poor spatial specificity and suboptimal control over dosage. Systemic administration often leads to inadequate biodistribution, off-target effects and a narrow therapeutic window. In this context, the development of targeted delivery platforms, such as nanoparticle-based carriers, represents a crucial step forward, enabling localized modulation of gasotransmitter activity within the TME while minimizing toxicity in healthy tissues (332, 333).

A complementary strategy involves targeting the biosynthetic enzymes responsible for gasotransmitter production, particularly in immunoregulatory myeloid cells. By selectively disrupting these immunosuppressive signaling in these cells, it may be possible to reprogram the TME toward a more pro-inflammatory and cytotoxic phenotype, thereby enhancing antitumor immunity without impairing systemic homeostasis (139). This immunologically focused strategy offers a pathway to improved therapeutic selectivity and the potential to synergize with existing immunotherapies.
